# Novel use of far UV-C light as a surface disinfectant in the poultry industry: trials on broiler farms show reduced bacterial burden in poultry anterooms and on wellington boots

**DOI:** 10.1186/s44364-026-00048-z

**Published:** 2026-10-08

**Authors:** Katharina Watson, C. Laudham, D. Francis, S. Daniels, S. Green

**Affiliations:** 1 Department of Agricultural Science and Practice, Royal Agricultural University, Stroud Road, Cirencester, GL7 6JS UK; 2 UV Care 222, School Street, Pontyclun, CF72 9FF UK; 3 Applied Group, Barker Lane, Chesterfield, S40 1DU UK

**Keywords:** Poultry, Biosecurity, Agriculture, Technology, Disinfection

## Abstract

Far ultraviolet-C (UV-C) irradiation at 222 nm has widely been shown to deactivate bacteria, viruses and fungi in in-vitro experiments. However, there are few studies testing the technology on real life surface. There are no peer reviewed studies on the efficacy and feasibility of far UV-C disinfection in real-life agricultural settings. The aim of this study was to investigate whether far UV-C irradiation installations on UK broiler poultry farms could reduce bacterial surface burden. On two farms one pair of wellington boots was submitted to far UV-C irradiation at 222 nm after staff exiting the bird housing, using a purpose build disinfection box. Swabs (*n* = 780) were taken from the bottom side of the soles before and after irradiation and from a pair of non-irradiated control boots. In a separate trial wall mounted Far UV-C systems were installed in a poultry anteroom and vestibule. Swabs (*n* = 615) were taken from irradiated and non-irradiated (control) anteroom floors divided into 4 zones (L1-4), vestibule floors divided into 2 zones (V1, V2) and walls divided into 2 zones (VW1, VW2) and the door handle (VD) leading to bird housing. For both studies surface swabs were taken twice a week during the first 4–5 weeks of a bird crop. This was repeated over 3–4 and 4–5 crops for the wellington trial and the poultry anteroom trial respectively. Swabs were plated in the laboratory and the number colony forming units (cfu’s) were determined. Overall, there was a significant cfu reduction between treatments (*p <* 0.001), after wellington boot exposure to far UV-C irradiation compared to pre-exposure cfu counts and cfu’s on control boots. Despite some technical challenges, across most anteroom and vestibule sampling locations, cfu’s from swabs taken from far UV-C irradiated areas were significantly lower than cfu’s from control area swabs. Far UV-C irradiation in livestock settings could be a promising added biosecurity layer on broiler farms and could be applicable to a range of livestock systems. Further research and development is required in areas such as installation adaptations to farms systems and settings, feasibility of implementation and cost benefit analyses.

**Supplementary Information:**

The online version contains supplementary material available at https://doi.org/10.1186/s44364-026-00048-z.

## Introduction

Animal disease outbreaks pose a significant threat to the poultry and the wider global farming sector, with far-reaching consequences for food security, public health, the economy, and international trade. The risks are expected to intensify with climate change, the rise of antimicrobial resistance (AMR), and evolving global trade patterns, which collectively increase the likelihood of endemic, epidemic and emerging disease threats, including the introduction of exotic pathogens [1]. In addition to the direct impacts on animal health and welfare, the economic burden associated with disease management and productivity losses is substantial [2]. There is a need to explore more robust layers of biosecurity and innovative, non-chemical approaches to disease prevention and control, particularly considering concerns regarding the reliance on chemical disinfectants. Evidence suggests that certain biocides used on farms may contribute to the selection and co-selection of antimicrobial resistance in farm pathogens such as *Escherichia coli*, typhoidal and non-typhoidal *Salmonella* serotypes. and Methicillin-resistant *Staphylococcus aureus* (MRSA) (3–8]. Moreover, the efficacy of chemical disinfection can be undermined by human error [9]. Novel tools that can be deployed across a range of sectors and production systems independent of drug resistance and less susceptible to human error are essential to addressing these challenges.

Ultraviolet C (UV-C) light, which operates at wavelengths between 100 and 280 nm, is widely employed as a biocidal agent for the disinfection of water, air, food, surfaces and medical equipment. Conventional germicidal UV-C systems typically emit UV radiation at 254 nm, where the radiation causes structural alterations to the DNA and RNA of microorganisms, resulting in DNA and RNA damages and hence pathogen inactivation [10]. However, UV-C radiation at these wavelengths poses well-documented risks to mammalian skin and eyes. Far UV-C light operates within the 200–230 nm range and has been proposed as a safer alternative. Its underlying mechanisms of action have been extensively described by Blatchley et al. [10]. Briefly, far-UVC light is generated by Krypton-Chloride (KrCl) excimer lamps and operates at the far end of the UVC spectrum (usually 222 nm wavelength), which has a more limited penetration depth than conventional UVC radiation at 254 nm. A substantial body of literature suggests that far UV-C exposure results in negligible or no damage to mammalian skin and eyes. This has been demonstrated in both animal models and human volunteer models (11–18], including for long-term exposure [15] although data remain limited. Numerous studies have reported that far UV-C achieves comparable pathogen inactivation efficiency to conventional 254 nm UV-C, although higher doses or longer exposure times may be required to attain equivalent disinfection efficacy [19, 20].

Far UV at 222 nm has shown to deactivate a range of bacteria relevant to animal and human health such as *S. aureus*, *E. coli*, *Clostridales* spp., *P. Aeruginosa* and *Bacillus *spp (21–27]. and can reduce the bacterial bioburden on real life surfaces [28]. It has also show to deactivate fungi [29–30] and viruses, including airborne viruses such as Sars-CoV-2 (COVID19) (31–35]. The dose required to deactivate varies by pathogen [24].

The majority of published researched is based on in-vitro studies where variables can be easily controlled. In vivo studies investigating the effect of environmental parameters and studies on real-life surfaces are sparse [22, 28, 36], highlighting the need to test far UV-C radiation via field studies on real-life surfaces.

Far UV-C technologies for disinfection in livestock agriculture have recently come to the market. These are primarily focused on water disinfection, with one Danish company advertising lamps for pig and poultry housing. However, field trial data is held by industry and to the authors knowledge there is no peer reviewed literature investigating far UV-C installation in a real-life livestock environment. In the poultry industry endemic infections such as *Salmonella* spp. and *Campylobacter* and epidemic diseases such as avian influenza (AI) cause persistent economic losses through reduced growth performance, increased mortality, flock depopulation. Highly pathogenic AI strains (e.g., H5N1, H5N8) also pose pandemic threats [37]. Therefore, this novel research aimed to test the efficacy of far-UV to reduce the bacterial bioburden in a poultry farm setting, firstly via a wellington boot disinfection box and secondly via installation of far UV-C lamps in poultry anterooms.

## Methods

### Ethical information compliance

Trials were approved by the Animal Welfare and Ethical Review Body (AWERB) of the Royal Agricultural University (Reference number 20242415-Watson). The trial complied with the Control of Artificial Optical Radiation at Work Regulations 2010, by ensuring that humans working around the installations were not exposed to far UV radiation. Animals were also not exposed to radiation during this trial. Krypton UV lights are not classed as medical devices and thus do not need approval from the Medicines and Healthcare Products Regulatory Agency (MHRA).

### Farm information

The trials took place between January 2023 and February 2025. Two commercial UK broiler farms (Farms A and B) where chosen for the study. Both farms housed Ross 308 broilers. Farm A was comprised of four bird sheds distributed over two floors and Farm B had two bird sheds in two different buildings. Farms represented a variety of UK chick suppliers, feed mills and processors, all were compliant with the Red Tractor farm assurance scheme and national law. Houses were stocked with 40,000 to 50,000 birds depending on the house size (Farm A: 3160 m^2^, Farm B: 2254m^2^), growing to a maximum stocking density of 38 kg/m^2^. Both farms operated a mixed sex, all-in all-out production system, with newly hatched chicks being placed into disinfected houses followed by a 37 to 42 day growth cycle (crop). Partial depopulation (thinning) occurred after week 4. All birds were housed in climate-controlled houses and kept on fresh wood shavings. Birds had *ad libitum* access to commercial broiler feeds, and water was continuously supplied via a storage tank connected to the main water supply. The farms record daily mortality, culling rates and bird weights as part of their day-to-day management, however, health data was not collected as part of this trial as a much longer study would be needed to show a relationship between use of far UV and bird health.

### Anterooms and vestibules: lay out and far UV installation

**Fig. 1:**
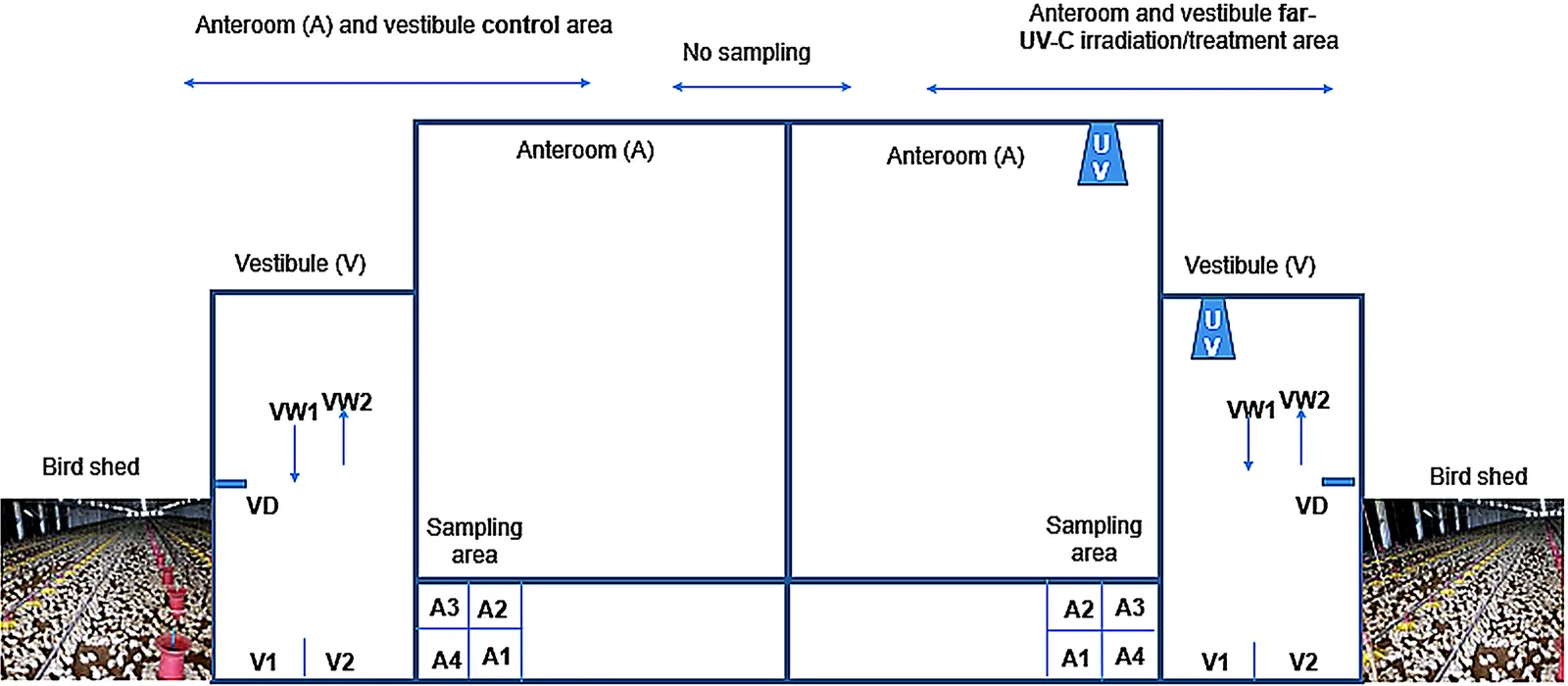
Broiler Poultry anteroom (A) and vestibule (V) experimental design of Far UV-C irradiated areas and control areas. Anteroom areas were divided into four 0.25m2 zones (A 1-4) with varying distance to the light source. Vestibules were divided into two 0.25m2 floor zones (V1, V2), two 0.25m2 wall zones (VW1, VW2) on opposite sides of the wall, and door handle (VD). The set of was mirrored in the adjacent control area, leaving a buffer of 2 meters between Far UV-C irradiated and control areas

Anteroom trials took place on farm A. The anteroom area is the common area outside a broiler house or houses where the boot disinfection process usually takes place. The vestibule area was the 3m^2^ area that connects the anteroom with the bird house. Anterooms were entered through an entrance bund where change of footwear took place. The second-floor anteroom area, with two vestibules leading to a bird shed each were divided into experimental (Far UV-C) and control areas (Fig. 1).

A bespoke designed wall/ceiling mounted far UV-C installation was designed (UV CARE 222 m Pontyclun UK) containing two Biolume™ UV-C excimer lamps (BiocareUV Ltd., Warrington, UK) enclosed in protective casing to protect from water and dust ingress. These were installed on the experimental side of anteroom area, while no changes were made to the other side of the anteroom (control). Lamps emitted radiation with a wavelength of 222 nm and irradiance at 0.5 m = 466mW/cm^2^ through one 400 mm x26mm Excimer Emitter Tube test tube. Lamps operated with an input power of 240 V/0.73 A and 150 W power. The far UV-C dose delivered was 34.04 mJ cm^− 2^. The first lamp was installed in the vestibule (See additional file 1 [A]). The unit was mounted with inbuild mounting brackets on the wall at a position that enabled Far UV radiation to irradiate the floor area. The distance between the far UV-C excimer lamp and the floor was 1.85 m. The second lamp was mounted in the anteroom close to the vestibule where it was fastened to the roof of the anteroom with suspension chains (Fig. 1, Additional file 1 [B]). The distance between the floor and the far UV-C excimer lamp was 1.90 m. The final lamp mounting heights were influenced by practicality and human safety aspects, while maintaining the highest possible irradiation power. Both unites were connected to the local 240 V AC supply and controlled using Passive Infrared (PIR) sensors, which were triggered when personnel entered the anteroom and vestibule areas. This ensured that humans were not exposed to far UV-C irradiation. At all other times, the Lume units were operating on a power duty cycle (2 minute on-1 min off) designed to deliver maximum irradiation to the floor of the anteroom area while maintaining the operating temperature of the lamp within the manufacturer’s specifications.

### Anteroom sampling protocol

Each replicate started after standard farm disinfection of the anterooms, vestibules and bird houses and arrival of a new crop of birds. Briefly, anterooms and vestibules were wet cleaned and disinfected using a 4-step disinfection procedure using Viroshield (2% volume per volume [v/v]), Ecofoam Advanced (2% v/v) and Kilcox extra (3% v/v) (All manufactured by Kilco, Lockerbie, UK) followed by formaldehyde disinfection with a thermal fogger. Anterooms and vestibules were also swept regularly during the crop cycle. Sampling started within three days of the birds arriving, depending on the farm-scheduled bird placing date. Anterooms and vestibules in Far UV-C irradiated areas and control areas were sampled twice weekly over the first four to five weeks of a bird crop resulting in 8–9 sampling events per crop. This was replicated over 5 non-consecutive bird crops in the anteroom area and 4 crops in vestibule area (Vestibule lamps failed after crop 4). On each sampling occasion environmental swab samples (TS/5–17 viscose tip swab, Technical Service Consultants Ltd, Heywood, United Kingdom) were collected from pre-defined zones (Fig. 1) using standard microbiological protocols for environmental swabbing (ISO 18593:2018, 38]. Sampling zones were marked with tape or pen to ensure consistency in swabbed surface size. Swabs were taken across the entire surface of the marked area by swabbing in a diagonal pattern, rotating the tip. Samples were suspended in Probact Amies Transport medium for transport (Technical Service Consultants Ltd, Heywood, United Kingdom).

### Wellington boot box installation

A bespoke “Boot Disinfection Box” (BDB) was designed (UV CARE 222, Pontyclun UK) to contain two pairs of protective boots (Additional file 2). The boots were held on a drawer mounted so that the soles of the boots were facing upwards and could slide in and out of the unit. It contained a single Lume Biolume™ UV-C excimer lamp (BiocareUV Ltd., Warrington, UK) (222 nm, irradiance at 0.5 m = 466mW/cm^2^) and was mounted so that the far UV-C irradiated the soles of the boots. The BDB was connected to the local 240 V AC supply. The box was lined with PTFE material to maximise the reflective capacity of the interior of the box to enable disinfection of the sides of the boots as well as the soles. Switches on the door ensured that the Far UV-C light was switched OFF when the lid is opened and only turned ON when the lid was closed. Wellington boots were irradiated for 2 minutes which avoided overheating, while ensuring maximum irradiation within the shortest practical time. The irradiation dose delivered during a 2-minute cycle was 108,733 mJ/cm^− 2^.

### Wellington boot box sampling protocols

Sampling protocols for farms A and B were identical. Two pairs of boots were used for the trial on each farm. One pair was subjected to far UV-C irradiation, the other acted as a control pair and did not undergo irradiation. Boots were assigned to two members of farm staff who wore them throughout the duration of each crop. Swab samples (TS/5–17 viscose tip swab, Technical Service Consultants Ltd, Heywood, United Kingdom) were taken after the morning bird health checks. Staff put on their boots when entering the anteroom area. On farm A boots were also dipped into a foot dip containing 1:200 concentrated Virkon^®^ S broad spectrum disinfectant (lanXESS, Trafford Park, Manchester, UK). All sampling took place upon return to the anteroom after exiting the bird house. Prior to swabbing all boots were shaken to dislodge larger litter particles. The far UV-C irradiated pair was swabbed at the bottom and the side of each wellington boot (left and right) before and after 5 min of irradiation in the BDB (Far UV-C irradiation at 222 nm). Control boots were only swabbed once, upon exiting the bird house. Boots were swapped using standard microbiological procedure. Researchers and farm staff were trained to swab the bottom of the soles and the side of the sole in a consistent manner using a rotating swab method. For soles, a horizontal and vertical sampling pattern was used covering the entire sampling area. For the side of the sole the swab was passed around the edge twice.

Swabs were suspended in Probact Amies Transport medium for transport (Technical Service Consultants Ltd, Heywood, United Kingdom).

### Laboratory analysis

All swabs were sent to a commercial laboratory at room temperature using the Royal mail special delivery service on the day of sampling to ensure samples arrived and were processed within 24 h of being taken. Samples were shipped in an insulated polystyrene box. CFU’s were determined using the total viable colony count method based on ISO 4833-1:2013 [39].

Briefly, swabs were processed vortexing for 60 s at 13,000 rpm to ensure transfer of organisms to the transport medium. The transport medium was then serial diluted to 10^− 6^. 1 ml was plated in triplicate and incubated under aerobic conditions at 30 °C for 72 h. Cfu’s were counted and multiplied by the dilution factor to obtain cfu/ml. The detection limit was 10 cfu’s/swab.

### Statistical analysis

All data was analysed using Genstat for Windows, 24th editions (VSN international 2022). The datasets were tested using the Shapiro-Wilk test which determined a normal distribution of all data. Subsequently, for BDB data, one-way Analysis of Variance (ANOVA) was used to test for statistical differences between log_10_ cfu counts between treatments (control boots, before Far UV-C irradiation cfu’s and post Far UV-C irradiation cfu’s), sites (Farms A and B), sampling location (sole or side of wellington boot) and boot (left/right wellington boot). Where a statistically significant difference was found, Tukey’s Honesty significant difference (HST) test (95%CL=upper) for multiple comparisons was used for pairwise comparisons. In order to understand differences between individual sampling zones, for the anteroom data, an independent two-sample t-test with group factor was used to test for statistical difference in cfu’s between Far UV-C irradiated areas (treatment) and control areas of the anteroom. Equality of variances was confirmed for all variables tested as part of the two-sample T-test. A Mann-Kendall test for monotonic trend was used to investigate accumulation of microbes in anterooms, vestibules and on wellington boots, throughout the bird crop.

## Results

### Wellington trial results

A total of 780 samples were collected for the trial. There was a significant difference between treatments (F[2] = 17.5, *p* = < 0.001, Table 1), which showed a reduction of cfu’s after wellington boot exposure to far UV-C-irradiation compared to pre-exposure cfu counts and cfu’s on control boots. Far UV-C irradiation reduced bacterial loads by 56% on average (Table 2). Furthermore, results showed that there was no interaction between the treatment variable and replicates, swabbing location (side/sole) and farms (A, B) (Table 1). Even though significant differences were found between replicates (F[8] = 1.20, *p* = < 0.001, Table 1) and Farms (F[1] = 9.42, *p* = < 0.01, Table 1), these did not affect the treatment variable. Swabbing on both the side and the sole of the boot reduced cfu’s significantly post irradiation, although there was a significant difference between locations (F[1] = 66.19, *p* = < 0.001, Table 1) with cfu’s from sole swabs being significantly higher and more inconsistent that cfu’s from swabs taken from the side of the boot. There was no difference between swabbing the left and right boots F[1] = 0.26, *p* = 0.607, with cfu’s reduced significantly in both after far UV-C exposure.

**Table 1:** One-way analysis of variance (ANOVA) showing the effects of far UV-C irradiation on log_10_ cfu’s on wellington boots between farms (A, B), replicates, left and right boots and sampling location (side/sole)

Variable	DF	F-value	Sum of squares	Mean square	Sig.
Treatment (*n* = 780)	2	17.5	69.807	34.904	*p* < 0.001
Location (side/sole)	1	66.91	69.811	122.846	*p* < 0.001
Side	2	8.08	34.653	17.326	*p* < 0.001
Sole	2	13.39	40.909	20.454	*p* < 0.001
Treatment*Location	2	1.57	5.750	2.875	0.210
Replicate	4	6.91	53.404	13.351	*p* < 0.001
Treatment*Replicate	8	1.2	18.573	2.322	0.295
Site (Farm A, B)	1	9.42	18.604	18.604	*p* < 0.01
Farm A	2	9.19	37.923	18.961	*p* < 0.001
Farm B	2	8.08	34.653	17.326	*p* < 0.001
Treatment*Site	2	0.33	1.3	0.65	0.720
Boot (Left/right)	1	0.26	0.528	0.528	0.607
Left boot	2	9.70	39.503	19.751	*p* < 0.001
Right boot	2	8.19	32.183	16.069	*p* < 0.001
Boot*Treatment	2	0.46	1.830	0.915	0.633

*Sig = Significance level at 95% CI

**Table 2:** Multiple comparison results showing level of statistical difference in log_10_ cfu’s on Far UV C-irradiated boots versus control boots and boot pairs pre irradiation

Variable	Control (*n* = 260)	Mean log_10_ cfu before Far UV-C (*n* = 260)	Mean log_10_ cfu After Far UV-C (*n* = 260)	SED* (before/after)	SED (before/control)	SED (after/ control)	Mean bacterial reduction (%)
Treatment (*n* = 780)	6.594^b^	6.604^b^	5.965^a^^	0.121	0.218	0.214	76%
Side (*n* = 390)	6.083 ^b^	6.300 ^b^	5.588^a^	0.173	0.163	0.167	80%
Sole (*n* = 390)	7.105 ^b^	6.907 ^b^	6.341^a^	0.131	0.171	0.162	73%
Farm A (*n* = 310)	6.437 ^b^	6.451 ^b^	5.705 ^a^	0.745	0.789	0.340	82%
Farm B (*n* = 470)	6.083 ^b^	6.300 ^b^	5.588 ^a^	0.228	0.308	0.324	81%
Left boot (*n* = 390)	6.636 ^b^	6.534 ^b^	5.915 ^a^	0.125	0.226	0.221	76%
Right boot (*n* = 390)	6.553 ^b^	6.674 ^b^	6.014 ^a^	0.234	0.325	0.263	78%

^Different superscripts indicate significant statistical difference at 95% CI determined by Tukey’s honest significant difference test. SED= Standard error of the difference

A significant accumulation of microbes was observed on farm B, in one replicate, showing a positive monotonic trend for all treatments (*p* < 0.01, Additional file 4), but no significant trends were identified in other replicates (Additional files 3 and 4).

### Anteroom and vestibule trial results

A total of 615 samples were analyzed from the anteroom and vestibule areas of farm B (control *n* = 303, UV *n* = 312, Table 3). Data from 5 non-consecutive bird crops/replicates were obtained for the anteroom area. The 5th replicate was abandoned in the vestibule area due to lamp failure.

Overall, across all sampling locations cfu’s from swabs taken from far UV-C irradiated areas were significantly lower than cfu’s from control area swabs. Analysis of individual sampling locations showed significantly lower cfu’s from all far UV-C irradiated areas (*p* < 0.05), except the anteroom and vestibule door handles and one statistical tendency for one area of the vestibule floor (V2, *p* < 0.1, Table 3). Analysis of statistical differences between replicates showed significantly lower cfu’s in far UV-C exposed areas compared to control areas in replicated 1,3,4 and 5, but differences in replicate 2 were not significant (data not shown).

**Table 3:** Two sample t-test results showing statistical significance levels in log_10_ cfu’s between swab samples taken from Far UV-C irradiated and control areas in poultry anterooms and vestibules

		N	Rep*	Mean	SD^	SEM^+^	SED~	t^#^	df^¬^	Sig’
**All samples**										
	Control	303	5	4.99	1.89	0.16	0.15	5.82	613	< 0.001
	Far UV-C	312		4.15	1.89	0.19				
**Anteroom** (all)										
	Control	175	5	5.22	1.81	0.14	0.19	4.19	353	< 0.001
	Far-UV-C	180		4.41	1.84	0.14				
Anteroom area 1 (A1)										
	Control	35	5	5.55	1.50	0.25	0.39	2.12	69	0.04
	Far UV-C	36		4.73	1.76	0.30				
Anteroom area 2 (A2)										
	Control	35	5	5.77	1.60	0.28	0.41	2.34	69	0.02
	Far UV-C	36		4.82	1.81	0.30				
Anteroom area 3 (A3)										
	Control	35	5	6.13	1.61	0.27	0.36	2.05	69	0.04
	Far UV-C	36		5.39	1.44	0.24				
Anteroom area 4 (A4)										
	Control	35	5	5.31	1.72	0.29	0.44	2.46	69	0.02
	Far UV-C	36		4.24	1.93	0.32				
Door handle (AD1)										
	Control	35	5	3.36	1.25	0.21	0.30	1.62	69	0.11
	Far UV-C	36		2.88	1.25	0.21				
**Vestibule (all)**										
	Control	128	4	4.68	1.94	0.17	0.24	4.16	258	< 0.001
	Far UV-C	132		3.70	1.87	0.16				
Vestibule floor 1 (V1)										
	Control	32	4	6.35	1.22	0.22	0.37	4.86	63	< 0.001
	Far UV-C	33		4.54	1.75	0.30				
Vestibule floor 2 (V2)										
	Control	32	4	5.61	1.51	0.27	0.46	1.91	63	0.06
	Far UV-C	33		4.73	2.13	0.37				
Vestibule wall (VW)										
	Control	32	4	3.23	1.40	0.25	0.33	2.45	63	0.017
	Far UV-C	33		2.42	1.28	0.22				
Vestibule door (VD)										
	Control	32	4	3.51	1.53	0.27	0.33	1.31	63	**0.194**
	Far UV-C	33		3.08	1.08	0.19				

Rep. * = number of replicates, SD^ =Standard deviation, SEM*= Standard error of the mean, SED ~ = Standard error of the difference, t^#^ =t-value, df^¬^=Degrees of Freedom, Sig’=Significance level and P-value (if non-significant)

A significant accumulation of microbes was observed on vestibule floors, in only one replicate, showing a positive monotonic trend (*p* < 0.05, Additional file 6). On vestibule walls, a positive monotonic trend was observed for the in 2/3 replicates, indicating a statistically significant accumulation of bacteria in control areas (*p* < 0.05), but not in far UV-C treated areas (Additional file 7). No significant monotonic trend was observed on anterooms floors (Additional file 5).

## Discussion

This novel research documents the first peer reviewed study investigating the use of far UV-C light at 222 nm as surface disinfection tool in a real-life agricultural setting.

Both the BDB and the poultry anteroom installation showed that far UV-C light can significantly reduce bacterial surface contamination. This aligns with the increased body of literature available from in vitro experiments showing that far UV-C light has the capacity to eliminate bacteria, viruses and fungi (e.g. 25, 26, 30, 32, 34) and the smaller body of literature documenting bacterial bioburden reduction on real-life surfaces and environments [22, 28, 40, 41]. Far UV-C irradiation is also a promising tool due to its safety advantages compared to UV-C irradiation at 245 nm. In this study, the installation of Passive Infrared (PIR) sensors meant that no humans were exposed to irradiation, even without detection of movement. This approach was also described by Memic et al. [40], who installed a wall mounted Far UV-C disinfection system in medical equipment rooms and could be a viable way to use bespoke far UV-C irradiation systems in human occupied spaces.

Although UV-C sterilisation cabinets are not a novel concept, to the authors knowledge the BDB was the first device build to specifically sterilize wellington boots using any kind of UV-C irradiation, hence specifically making it relevant for a range of agricultural applications or applications in any sectors that rely on biosecurity. Even the more advanced boot disinfection systems in agricultural settings rely on chemical disinfection involving a washing process, which may be prone to human error [9]. Foot dips require regular changing and appropriate chemical dosing, so a simple on-off switch system could help mitigate management errors [42].

Disinfection of footwear is a vital biosecurity measure in poultry farming, limiting the introduction of infectious disease agents [43]. Although here we piloted the BDB on poultry farms, the unit is transferable to other livestock farming systems and beyond. Chemical disinfection via footbaths, although considered essential, does not completely eliminate viable pathogens on footwear [44, 45]. Hauck et al. [44] showed that both highly and low pathogenic avian influenza survived for 72 h in quaternary ammonia-based footbaths and Rabie et al. [45] conclude that boot dips in poultry farms are frequently ineffective when they measured *Salmoella* presence in boot dip samples.n

Results from the BDB show that’s cfu’s on UV irradiated wellington boots (bottom and side of soles) were significantly lower than those on control boots and boot pairs pre-irradiation. The average bacterial reduction ranged from 73% − 82% of boot pairs after far UV-C exposure, equalling to an average of 0.5 log reduction. A study by Nasr et al. [46] tested five disinfectants on rubber boots inoculated with poultry litter. They report a viable colony count log reduction after footbath exposure of 0.79 to 4.17 on day 1 and 0.63 to 2.51 om day 2, depending on disinfectants used. This may suggest that chemical footbaths are more efficient than far UV-C irradiation. However, it is difficult to draw meaningful conclusions when comparing our results to in vitro studies or more controlled experimental set ups.

No technical issues where experienced with the BDB throughout the duration of the trial. A recommendation would be to design a system that allows for removal of litter and faeces prior to far UV-C irradiation. It is likely, that presence of litter on boots will have affected the ability of the far UV-C to deactivate pathogens [44]. Staff did shake the boots to remove larger pieces of litter, but did not specifically clean the soles of the boots. Generally, disinfectants lose efficacy in the presence of organic materials such as faeces, bedding, and dirt [43, 44]. There is also evidence that far UV-C does not penetrate poultry litter effectively. Memic et al. [41] showed a reduced effectiveness of far UV-C irradiation in deactivating MRSA and a viral bacteriophage after inoculation in poultry manure. The presence of a thick litter layer on boots could explain the more inconsistent results from sampling the bottom of soles and the higher number of cfu’s compared to the cfu’s on the much cleaner sides of the sole.

The far UV-C poultry anteroom installations also showed promising results. Poultry anterooms and vestibules play a significant role in the transfer of pathogens in poultry housing, as do entrance areas to animal housing in other livestock systems, specifically indoor systems that rely on strict entry and exit biosecurity procedures. Here we show a significant reduction of bacterial burden in most far UV-C irradiated areas compared to control areas. The lack of statistical significance for door handles and one vestibule floor areas may be attributed to limited reach of the far UV-C irradiation. For example, the anteroom door handles were a longer distance away from the light source than other anteroom areas and the irradiation area of the vestibule floor (V2 only) was obstructed by a wash basin. Areas that are not in direct reach of the far UV-C irradiation will likely be present in any on farm ceiling installation. In future applications, this should be considered during standard cleaning and disinfection procedures. It is also unclear why no significant differences in bacteria burden were observed in one replicate, as we could not link this to any management changes or other on-farm events.

It is challenging to draw meaningful comparisons to other studies in terms of disinfection efficiency. The irradiation dose in anterooms and vestibules was 34.04 mJ cm^− 2^ per 2-minute cycle and we show that the bacterial burden in far UV-C irradiated areas was on average 0.85 log lower than in control areas. Similar set ups did not compare to a control area and calculated the percentage of reduction before and after exposure. To the authors knowledge only a couple of studies investigated far UV-C installation in room sized areas. Buonanno et al. [35] showed a 99.8% reduction in airborne murine norovirus in a mouse cleaning room, when irradiated at a peak dose of 79.5 mJ cm^− 2^. Eadie et al. [22] achieved a 92% reduction on *Staphylococcus aureus* over an 8-hour exposure at 23 mJ cm^− 2^. Although both studies are more alike to in vivo experiments, areas tested were likely not exposed to the same level of contamination. In addition, they investigated specific pathogens rather than investigating bacterial surface burden.

Here were showed the potential for developing real life far UV-C irradiation systems in livestock settings. However, despite designing protective casings for anteroom and vestibule, we did experience failure due to dust ingress. Our technical issues suggest that a more robust system would be needed for a permanent installation. Any realistic wall-mounted installation would need to be developed to withstand the farm environments and generated substances such as water and dust. Future research should also consider whether dust removal could be integrated in a far UV-C disinfection system. In addition to being damaging to technical equipment, dust can act as a carrier of microorganisms. Moreover, bacterial counts in settled poultry house dust have been shown to increase over time [47].

Permanent installations would also rely on a stable electrical supply, which is not always available on farms. Finally, ongoing technical support would have to be available for the set up and running of the system. Bespoke installations could also increase the efficacy of the system. Here we used commercially available lamps with non-adjustable irradiance levels that had to be hung at a safe height, while taking other practicalities into account. It is therefore likely that efficacy could be improved through bespoke commercial installations.

Recommendation for further research also include social science studies to investigate opportunities and barriers to install far UV-C systems on farms. Although this was not part of our study design feedback from farmers suggested that both systems, especially the BDB were easy to use, did not significantly impact workload, with a consensus that they would install these systems on the broiler farms, if there was a clear economic benefit. There is however no research available yet of the long-term effect off far UV-C installations that aim to disinfect the environment for humans and animals health. Long term studies on several farms would be needed to show a clear association between disinfection measures and occurrence of disease. In this study, we also only investigated bacterial bioburden without testing disinfection efficacy of specific pathogens. Far UV-C has shown efficacy against a range of pathogens, including those relevant to livestock farming. However, more data on the ability of far UV-C light to reduce specific farm-relevant pathogens or pathogen indicator organisms in a real-life farm setting would be useful.

Although a significant reduction in microorganism was achieved for many tested sampling areas, it is important to discuss to what degree these findings are biologically relevant. The bacterial surface reductions observed are too low to be classed as successful disinfection which requires a 4–6 log reduction equalling a 99.9% to 99.9999% pathogen reduction [48]. Here we show an average log reduction of 0.5. Future research and development should address how the effectiveness of far UV-C installations can be improved for use in commercial farming systems. Unless successful disinfection is achieved, the replacement of chemical disinfection is unlikely. Far UV-C and chemical disinfection combined may however be a useful tool to improve pathogen burdens on broiler farms. Despite the abovementioned limitations, we present a promising tool for surface disinfection that could be applied to a range of livestock and wider agricultural settings. This technology could be an innovative on-farm solution for preventing infectious disease on farms through the addition of an extra biosecurity layer, hence addressing global concerns around human and animal health and food security.

## Supplementary Information

Below is the link to the electronic supplementary material.

Supplementary Material 1: Additional file 1: UV-C krypton-chlorine (Kr-Cl) excimer lamps installed in the anteroom area of the poultry housing adjacent to the vestibule are (A), and the vestibule (B) leading to the bird housing.

Supplementary Material 2: Additional file 2 (.mp4): Video file of wellington boot disinfection box

Supplementary Material 3: Additional 3: Figure showing accumulation of bacterial loads over time on wellington boots on farm A.

Supplementary Material 4: Additional 4: Figure showing accumulation of bacterial loads over time on wellington boots on farm B.

Supplementary Material 5: Additional 5: Figure showing accumulation of bacterial loads over time on anteroom floors

Supplementary Material 6: Additional 6: Figure showing accumulation of bacterial loads over time on vestibule floors

Supplementary Material 7: Additional 5: Figure showing accumulation of bacterial loads over time on vestibule walls.

## Data Availability

The datasets used and/or analysed during the current study are available from the corresponding author on reasonable request.
