# Supplementary material for: Novel use of far UV-C light as a surface disinfectant in the poultry industry: trials on broiler farms show reduced bacterial burden in poultry anterooms and on wellington boots
Source: Poult Sci Manag. 2026 Oct 8;3(1):25. doi: 10.1186/s44364-026-00048-z (PMC13649860; doi:10.1186/s44364-026-00048-z)
Supplement: Supplementary file 4 [file 44364_2026_48_MOESM6_ESM.pdf]

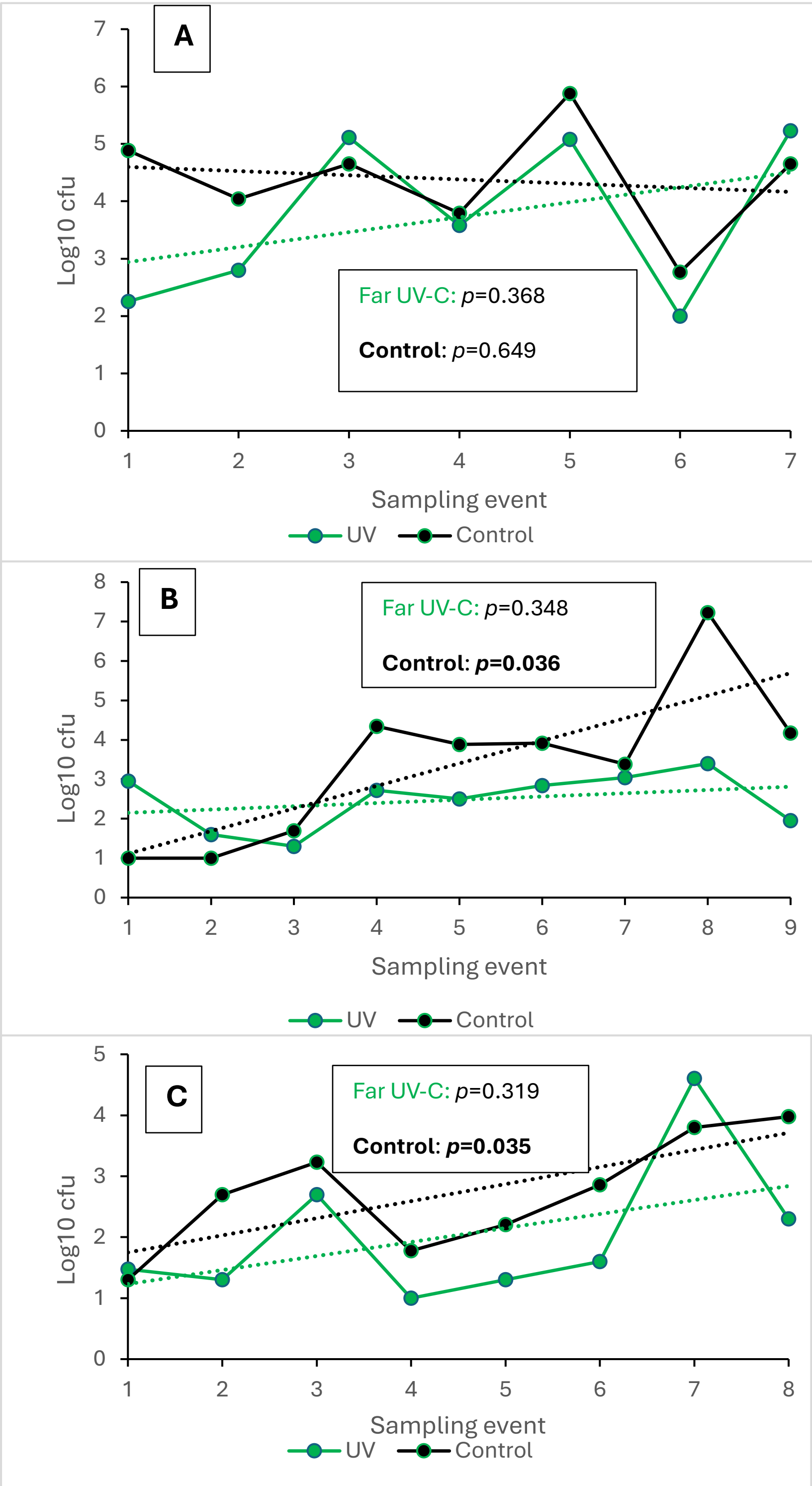

Additional file 7: Figure showing the accumulation of bacterial burden on vestibule walls. Wall areas 1and 2 were combined for analysis. The central box shows the *p*-values for each variable after analysis of monotonic trend using the Mann-Kendall test. Statistically significant values are indicated in **bold.** A=replicate 1, B=replicate 2, C-replicate 3.
