# Supplementary material for: Novel use of far UV-C light as a surface disinfectant in the poultry industry: trials on broiler farms show reduced bacterial burden in poultry anterooms and on wellington boots
Source: Poult Sci Manag. 2026 Oct 8;3(1):25. doi: 10.1186/s44364-026-00048-z (PMC13649860; doi:10.1186/s44364-026-00048-z)
Supplement: Supplementary file 5 [file 44364_2026_48_MOESM7_ESM.pdf]

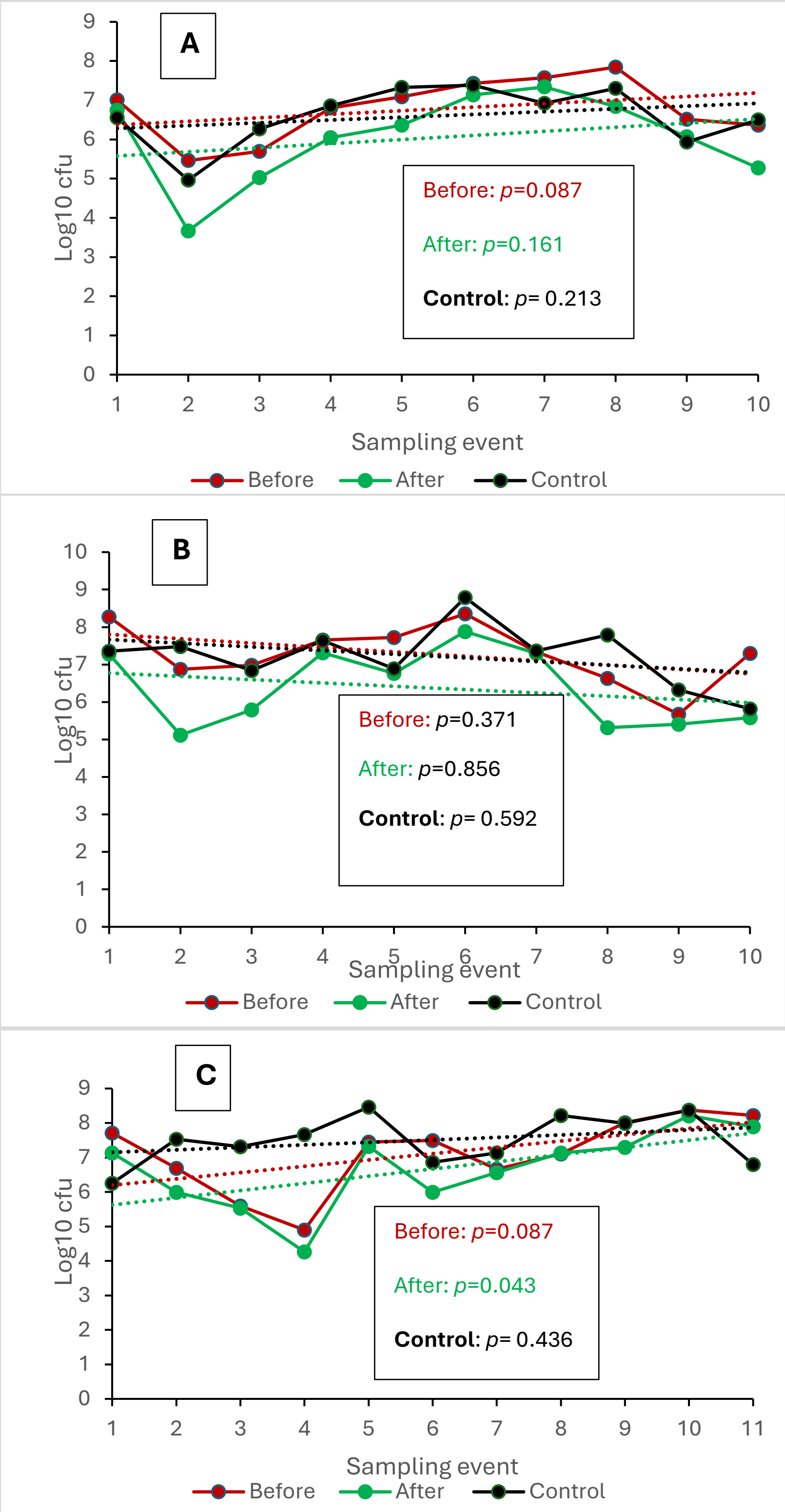

Additional file 3: Figure showing the accumulation of bacterial burden on wellington boots on Farm A. The central box shows the *p*-values for each variable after analysis of monotonic trend using the Mann-Kendall test. A=replicate 1, B=replicate 2, C-replicate 3. The variables “Side”, “Sole”, “left” and “right” were merged for analysis.

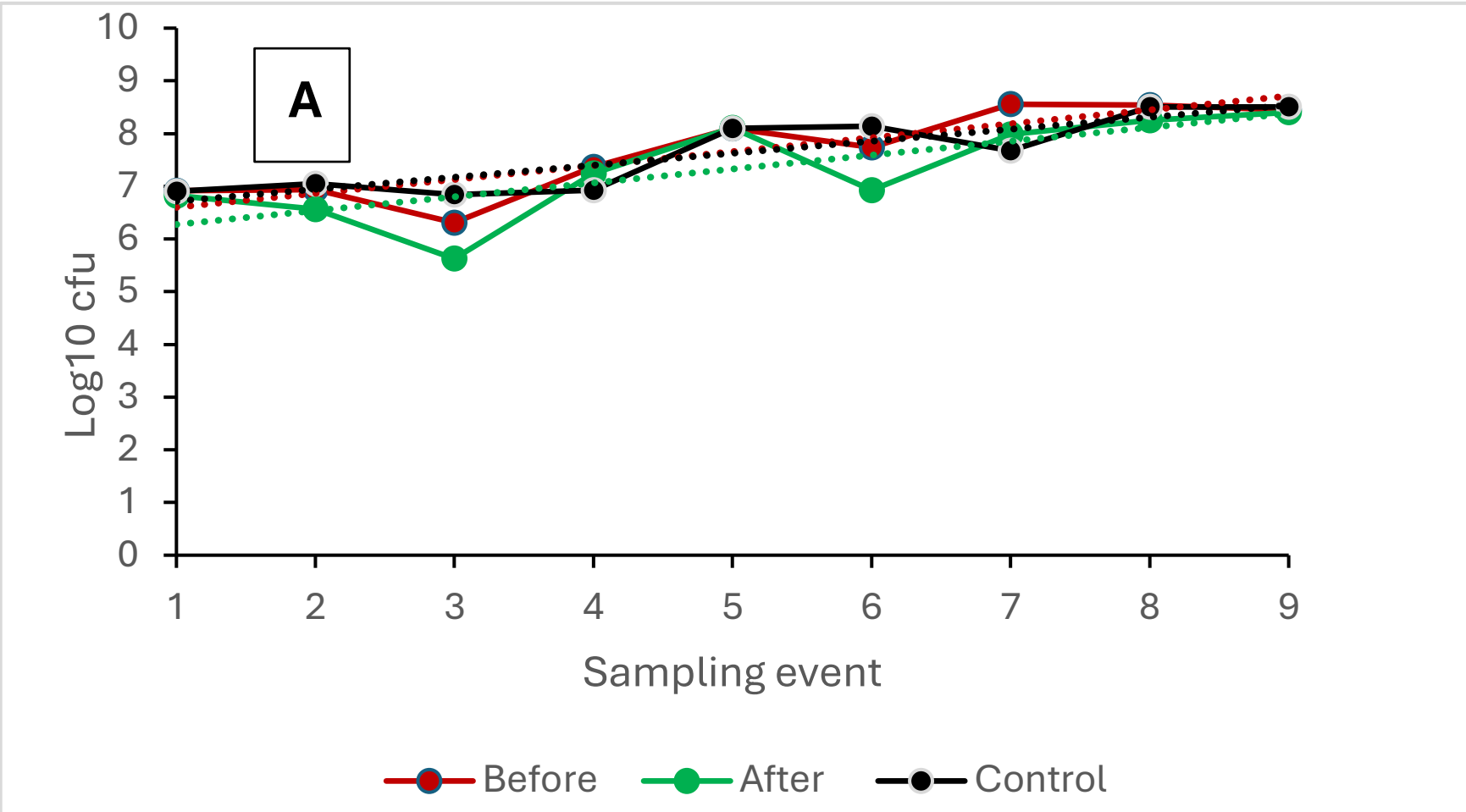
A
10
9
8
7
6
5
4
3
2
1
0
Log10 cfu
1
2
3
4
5
6
7
8
9
Sampling event
Before
After
Control
