# Supplementary material for: Novel use of far UV-C light as a surface disinfectant in the poultry industry: trials on broiler farms show reduced bacterial burden in poultry anterooms and on wellington boots
Source: Poult Sci Manag. 2026 Oct 8;3(1):25. doi: 10.1186/s44364-026-00048-z (PMC13649860; doi:10.1186/s44364-026-00048-z)
Supplement: Supplementary file 6 [file 44364_2026_48_MOESM1_ESM.docx]

A

B

Additional file 1: UV-C krypton-chlorine (Kr-Cl) excimer lamps installed in the lobby area of the poultry housing adjacent to the vestibule are (A), and the vestibule (B) leading to the bird housing.
